# Monitoring Meat Freshness with Intelligent Colorimetric Labels Containing Red Cabbage Anthocyanins Copigmented with Gelatin and Gallic Acid

**DOI:** 10.3390/foods13213464

**Published:** 2024-10-29

**Authors:** Minyoung Kwak, Sea C. Min

**Affiliations:** Department of Food Science and Technology, Seoul Women’s University, 621 Hwarang-ro, Nowon-gu, Seoul 01797, Republic of Korea

**Keywords:** beef, copigmentation, freshness indicator, pH, squid, total volatile basic nitrogen

## Abstract

Polyvinyl alcohol (PVA)-based pH-responsive color indicators were developed using red cabbage anthocyanin (Anth) copigmented with gelatin and gallic acid (GA). The indicator prepared with gelatin and GA (GA/gelatin/Anth/PVA) was highly resistant to light exposure. GA/gelatin/Anth/PVA exhibited distinct color changes in pH 2–11 buffer solutions and stable color indication in acidic and neutral solid systems (pH 2 and 7) at 97% relative humidity. GA/gelatin/Anth/PVA exhibited the highest sensitivity to dimethylamine, followed by ammonia and trimethylamine. The addition of gelatin and GA facilitated hydrogen bonding, which enhanced thermal stability and water solubility without compromising tensile properties. A color change from purple to blue signaled spoilage when total volatile basic nitrogen values for beef and squid reached 21.0 and 37.8 mg/100 g, respectively. The GA/gelatin/Anth/PVA indicator shows potential for indicating the freshness of raw beef.

## 1. Introduction

According to the Food and Agriculture Organization of the United Nations, approximately 1.3 billion tons of food are discarded globally each year [1]. Consumers rely strongly on food date labels to assess food stability, discarding or avoiding purchases beyond the specified date, even if the food remains unspoiled. Freshness indicators can mitigate this problem of discarding unspoiled food. These indicators respond to compounds generated with an increase in food storage time, such as carbon dioxide, oxygen, volatile nitrogen compounds, and hydrogen sulfide, and display real-time color changes that promptly inform consumers about the freshness of the food [2]. Consumers can check the quality of the food in real time by examining the freshness indicator attached to the food. This type of intelligent packaging that interacts deliberately with the food or its environment can minimize food wastage by offering valid freshness information.

Colorimetric pH indicators are important in the food supply chain because they can detect pH changes caused by chemical reactions or microbial growth. As the spoilage of many food products directly correlates with alterations in pH, employing colorimetric indicators is an attractive alternative for consumers to assess the quality of those products in real time [3]. During spoilage, the pH of the meat increases as the amino acids of the constituent proteins are hydrolyzed into alkaline compounds, including volatile amines, such as trimethylamine (TMA), dimethylamine (DMA), and ammonia (NH_3_) [4].

Anthocyanins (Anths) can react with gases generated during food spoilage, and they exhibit different chemical structures and varying colors depending on the pH. Various Anth-based pH indicators have been developed to convey real-time freshness to consumers through color changes [5]. Anths extracted from natural sources, including rose, mulberry, roselle, black rice bran, and red barberry, and a polymer or mixture of polymers, including starch, chitosan, polyvinyl alcohol (PVA), microcrystalline cellulose, and gelatin, have been widely used in the development of food freshness indicators. These indicators are employed for monitoring pH changes in a variety of foods, such as shrimp, mitten crab, chicken tenderloins, and fish fillets [6,7,8,9,10].

Owing to the instability of the flavylium cation, Anths are susceptible to various factors, including light exposure, pH, temperature, and solvents. Such conditions can lead to the degradation of Anths, affecting their color and functionality, thereby limiting their utility as a colorant in food quality indicators. This disadvantage can be addressed through a process known as “copigmentation”, which involves the interaction of Anths with “copigments” to stabilize their structure. Copigments can be categorized into three types—biopolymer, phenolics, and metal ions [11].

The Anth content was reported to be approximately 1.7-times higher in a solution with guar gum added after a 10-day storage period at 40 °C than that in a solution without the addition of guar gum [12]. In another study, a 1.8-fold-higher Anth content was found in wine upon the addition of a phenolic compound, gallic acid (GA), than that in wine without GA following a 6-month maturation period [13]. The Anth content of blueberry juice increased by approximately 1.2-fold upon the addition of GA compared with that in juice without GA following a 10-day storage period at 40 °C [14]. These studies indicate that GA effectively stabilizes the Anth structure. Moreover, the simultaneous use of different types of copigments to further enhance the stability of Anths has been explored. Chen et al. [15] showed that incorporating rutin and whey protein isolate (WPI) as copigments for mulberry Anths resulted in greater thermal stability than that achieved by incorporating rutin alone. This enhanced stability was attributed to the hydrophobic interaction between Anths, rutin, and β-lactoglobulin in WPI. Similarly, Tan et al. [16] reported that employing pectin and heated WPI as copigments for blueberry Anths led to an approximately 1.4-fold-higher retention efficiency than that obtained using heated WPI alone after a 35-day storage period at 4 °C. This improvement was attributed to the hydrophobic interaction, electrostatic interaction, and hydrogen bonding between pectin and Anths, all of which enhanced Anth stability.

In light of the abovementioned context, the present study was conducted with the following objectives: (1) To develop pH indicators containing Anths with high stability using copigments, namely gelatin and GA; (2) To investigate the effects of light, temperature, and relative humidity (RH) on the functioning of pH indicators; (3) To evaluate the sensitivity of pH indicators to representative volatile amines (TMA, DMA, and NH_3_) produced during protein food spoilage; (4) To determine the physical properties, including morphology, surface chemistry, thermal stability, tensile properties, and water solubility of the fabricated pH indicators; and (5) To verify the potential application of the novel indicators for testing raw beef stored at 4 and 25 °C and raw squid stored at 4 °C. The novelty of this study lies in its evaluation of the stability of pH indicators with Anths using a biopolymer (gelatin), a phenolic compound (GA), and a metal ion (aluminum ion; AL) as copigments, either individually or in combination, to determine the optimal copigment composition that provides high stability. Additionally, this study uniquely assesses the indication stability of the pH indicator under various environmental conditions of light, temperature, and RH.

## 2. Materials and Methods

### 2.1. Materials

Red cabbage Anth extract powder (Anth content: 1685.75 ± 10.16 mg/100 g), which was used as the dye material for the pH indicator films, was purchased from ES food (Gunpo, Republic of Korea). PVA (Mw = 146,000–186,000 g/mol, >99% hydrolyzed), glycerol, GA, and aluminum chloride hexahydrate were purchased from Sigma-Aldrich (St. Louis, MO, USA); gelatin (Mw = 50,000–100,000 g/mol) was purchased from Jeongwoodang (Seoul, Republic of Korea), and 0.5 M hydrochloric acid (HCl) solution was purchased from Samchun Pure Chemical (Pyeongtaek, Republic of Korea). TMA, DMA, and NH_3_ were purchased from Sigma-Aldrich. Raw beef (flat-iron steak) and raw squid (body) for the monitoring of freshness were purchased from E-mart (Seoul, Republic of Korea). The raw beef and squid were stored at 4 °C for up to 24 h prior to being used in experiments.

### 2.2. Fabrication of pH Indicator Films

Depending on the combination of the selected copigments—gelatin, GA, and AL—six pH indicator films were fabricated. To fabricate the PVA-based pH indicator film (Anth/PVA) without copigments, 50 g of PVA solution (9.9%, *w*/*w*) and 50 g of distilled water (DW) were mixed and placed on a stirrer (analog magnetic stirrer with hotplate; Misung Scientific Co., Ltd., Seoul, Republic of Korea) for 1 h. Next, 1 g of glycerol and 0.045 g of Anth were added, and after shaking for 1 h, as described above, an Anth/PVA film-forming solution was prepared.

For fabricating the PVA-based pH indicator film with the copigment gelatin (Gelatin/Anth/PVA), the method described by Zeng et al. [17] to produce pH indicators using mulberry Anths was followed. First, 50 g PVA solution (10%, *w*/*w*) was mixed with 50 g gelatin solution (2%, *w*/*w*), and after shaking for 1 h on a stirrer at approximately 300 rpm, 1 g of glycerol and 0.045 g of Anth were added. The mixture was shaken at approximately 300 rpm for 1 h to prepare a gelatin/Anth/PVA film-forming solution.

For preparing the PVA-based pH indicator film incorporating the copigment GA (GA/Anth/PVA), GA (0.045 g) was dissolved in 95% ethanol (0.450 g), and this was added to the Anth/PVA film-forming solution (101.045 g). A high concentration of ethanol was used to promote dissolution of GA in the film-forming solution and thus prevent the aggregation of GA during the process of film formation [18]; the mixture was shaken for 30 min to prepare a GA/Anth/PVA film-forming solution.

For fabricating the PVA-based pH indicator film incorporating the copigments gelatin and GA (GA/gelatin/Anth/PVA), the solution of GA (0.045 g) prepared with 95% ethanol (0.450 g) was mixed with the gelatin/Anth/PVA film-forming solution (101.045 g) and the mixture was shaken for 30 min to prepare a GA/gelatin/Anth/PVA film-forming solution.

For preparing the PVA-based pH indicator film incorporating the copigments gelatin and AL (AL/gelatin/Anth/PVA), AL (0.009 g) was added to the gelatin/Anth/PVA film-forming solution (101.045 g); the mixture was shaken for 30 min to prepare an AL/gelatin/Anth/PVA film-forming solution.

For fabricating the PVA-based pH indicator film incorporating the copigments gelatin, GA, and AL (AL/GA/gelatin/Anth/PVA), 0.045 g of GA was dissolved in 0.450 g of 95% ethanol, and this solution was mixed with 101.045 g of the gelatin/Anth/PVA film-forming solution for 30 min. Thereafter, 0.009 g of AL was added, and the mixture was shaken for 30 min to prepare an AL/GA/gelatin/Anth/PVA film-forming solution.

The pH of all the film-forming solutions was adjusted to 4 using a 0.5 M HCl solution. At this pH, Anths exhibit high color stability [19]. To achieve a film thickness of 0.1 mm based on the solid content, casting of approximately 16.8, 14.3, 16.6, 14.3, 14.3, and 14.3 g of Anth/PVA, Gelatin/Anth/PVA, GA/Anth/PVA, GA/gelatin/Anth/PVA, AL/gelatin/Anth/PVA, and AL/GA/gelatin/Anth/PVA film-forming solutions, respectively, was carried out in respective Petri dishes (90 mm). The cast films were left to dry naturally for 48 h at 25 °C in a dark room.

### 2.3. Color Stability of pH Indicator Films Against Fluorescent Light

The color stability was evaluated following the method of Zhang et al. [20]. The fabricated Anth/PVA, Gelatin/Anth/PVA, GA/Anth/PVA, GA/gelatin/Anth/PVA, AL/gelatin/Anth/PVA, and AL/GA/gelatin/Anth/PVA indicators were stored in a constant-humidity box at 50 ± 5% RH for 24 h. Next, each film was cut up into 2 × 2 cm pieces to measure the color values on day 0 (L*_0_, a*_0_, and b*_0_) using a colorimeter (Minolta Chroma Meter CR-400; Minolta Camera Co., LTD., Osaka, Japan). To calibrate the calorimeter, a white standard plate (Minolta calibration plate No. 14233126; Y = 87.4, x = 0.3174, and y = 0.3353) was used. The sensor side of the colorimeter was placed on the film at the coordinates of CIELab (L*, a*, and b* values), and the color values were measured with the illuminant D65 and 2° standard observer. The L*, a*, and b* values represent lightness, deviation in color from green to red, and deviation in color from blue to yellow, respectively [21]. The six fabricated indicators were stored at 4 °C (75% RH) for 12 days under the fluorescent-lamp (Samjung, Hwaseong, Republic of Korea; wavelength range: 580–600 nm) condition, as well as under the dark-room condition (in an incubator). The light intensity of the lamp was 1058.65 ± 88.17 lux, and the distance between the indicator film and the fluorescent lamp was 40 cm. During the storage period, the color values (L*, a*, and b*) were measured at 48 h intervals, and the color difference (∆*E*) was calculated using the following equation [22]:

(1) $$\Delta E=\sqrt{{\left(L^{*}-L_{0}^{*}\right)}^{2}+{\left(a^{*}-a_{0}^{*}\right)}^{2}+{\left(b^{*}-b_{0}^{*}\right)}^{2}}$$

### 2.4. pH-Dependent Change in the Color of the Anth Solution and GA/Gelatin/Anth/PVA

For analyzing color changes in the Anth solution at different pH levels, an Anth solution was prepared by adding 0.2 g of Anth to 100 mL of DW and stirring for 10 min. Thereafter, 4 mL of the Anth solution was mixed with 1 mL of pH buffer solution (pH 2–11) [23]. The UV–Vis spectra were analyzed with a microplate spectrophotometer (SpectraMax M2; Molecular Devices, San Jose, CA, USA) over a wavelength range of 400–700 nm [24].

To analyze the changes in the color of GA/gelatin/Anth/PVA at different pH levels, the GA/gelatin/Anth/PVA films were cut up into 2 × 2 cm pieces and placed in buffer solutions of pH 2–11 for 10 min. The indicators were then taken out of the buffer solutions, and any excess buffer solution was removed with a wipe (Kimtech Science Wiper; Yuhan-Kimberly, Seoul, Republic of Korea). The color values of the indicators were determined under fluorescent light following the procedure described in Section 2.3.

### 2.5. Determination of the Limit of Detection (LOD) and Limit of Quantification (LOQ) Regarding Volatile Amines for GA/Gelatin/Anth/PVA

The LOD is the minimal concentration of the target material that can be detected, whereas the LOQ represents the minimal concentration of the target material that can be reliably analyzed [25]. The LOD and LOQ of GA/gelatin/Anth/PVA for TMA, DMA, and NH_3_ were estimated using the slope (S) and standard deviation (σ) of the calibration curve, calculated as 3.3 σ/S and 10 σ/S, respectively [26]. Initially, the color of GA/gelatin/Anth/PVA (2 × 2 cm) was measured using a colorimeter. Thereafter, the film was affixed to the inner side of the lid of a Petri dish (90 mm). The TMA solution at concentrations of 500, 700, 850, and 1000 mg/L, DMA solutions at concentrations of 60, 80, 100, and 120 mg/L, and NH_3_ solution at concentrations of 60, 100, 120, and 150 mg/L were each applied in 15 mL quantities to the Petri dish. The lid and plate of the Petri dish were sealed using parafilm, and the assembly was stored for 12 h at 22 ± 2 °C. Subsequently, the color of the indicator was measured as previously described, and a calibration curve was constructed using the concentrations of TMA, DMA, and NH_3_, along with the corresponding ∆*E* values of the indicator.

### 2.6. Storage of GA/Gelatin/Anth/PVA in Controlled pH, RH, and Temperature Conditions

WPI films set to pH 2, 7, or 11 were used to adjust the pH of GA/gelatin/Anth/PVA. The WPI film was prepared following the method described by Sothornvit et al. [27]. A 10% (*w*/*w*) solution of Hilmar 9410 (Hilmar Ingredients, Hilmar, CA, USA) was mixed with 5% (*w*/*w*) glycerol and shaken for 20 min. Subsequently, the WPI film-forming solution was obtained after 20 min of heating at 85 °C and subsequent cooling. The pH of the film-forming solution was adjusted to 2, 7, and 11 using 0.5 M HCl solution or 0.5 M NaOH solution (Samchun Pure Chemical) before casting onto the Petri dish (90 mm). To achieve a film thickness of approximately 0.1 mm for a pH of 7, approximately 6.7 g of the solution should be applied to the Petri dish. After drying for 48 h at 25 °C, GA/gelatin/Anth/PVA (2 × 2 cm) was placed on the WPI film (4 × 4 cm) at pH 2, 7, and 11 to match the pH of the indicator with that of the WPI film. The GA/gelatin/Anth/PVA on the WPI film was stored at 23 ± 2 °C for 24 h, after which the pH of the indicator film was confirmed using a pH meter (Apera pH 60; Apera Instruments, Wuppertal, Germany), capable of measuring the pH of films. Next, the indicator films set at pH 2, 7, and 11 were placed in desiccators set at 74, 84, and 97% RH using sodium nitrate, ammonium sulfate, and potassium sulfate (Samchun Pure Chemical), respectively [28]. The desiccator was stored in a shaded place inside a refrigerator (Samsung, Seoul, Republic of Korea) or an incubator (JSR, Gonju, Republic of Korea) at 4 ± 1, 10 ± 2, and 25 ± 2 °C for 4 days. The color of the film was measured using a colorimeter at 24 h intervals for 4 days. The ∆*E* values were calculated using the same method as described in Section 2.3 for the color stability of pH indicator films against fluorescent light.

### 2.7. Film Characterization

Scanning electron microscopy (SEM), fourier-transform infrared spectra (FTIR), thermal stability, tensile properties, and water solubility were determined to investigate the effects of Anth, gelatin, and GA incorporation on the properties of the PVA-based films.

PVA, Anth/PVA, and GA/gelatin/Anth/PVA films (2 × 2 cm) were platinum-coated and morphologically analyzed using SEM (Teneo VS, Hillsboro, OR, USA) at 10 kV to examine the surface and cross-sections of each film [29].

To analyze the chemical bonding on the film surface, the Nicolet iS10 FTIR spectrometer (Thermo Scientific, Waltham, MA, USA) was used. The range of frequency was 400–4000 cm^−1^ at a constant resolution of 4 cm^−1^. The attenuated total reflectance was used to analyze the properties of the surface chemical compounds [30].

Thermal stability was assessed via thermogravimetric analysis (TGA) using an SDT Q600 instrument (TA Instruments, New Castle, DE, USA). Nitrogen (N_2_) was used as the carrier gas, and the temperature was ramped at a rate of 10 °C/10 min from 25 to 600 °C [31].

For tensile properties, indicator films sized 50 × 8 mm were stored in a desiccator (22 ± 2 °C/50 ± 2%) for 48 h before testing. Tensile testing was performed using a tensile tester (Withlab Co., Ltd., Anyang, Republic of Korea), following the standard D882-01 method [32]. The grip distance and load rate were set at 50 mm and 50 mm/min, respectively.

Water solubility was determined following the method of Liu et al. [33]. Films (2 × 2 cm) were dried in an oven at 105 °C for 24 h, and their weights (*W*_1_) were measured. The films were then placed in 50 mL DW at 23 ± 2 °C for 24 h, shaken at 100 rpm, dewetted using a wipe, and dried again in an oven at 105 °C for 24 h. Subsequently, the weight (*W*_2_) was measured, and the water solubility was calculated using the following Equation (2):

(2) $$Water\;solubility\;\left(\%\right)=\left(\frac{W_{2}-W_{1}}{W_{1}}\right)\times 100$$

### 2.8. Freshness Monitoring of Beef and Squid Using GA/Gelatin/Anth/PVA

Beef and squid body samples were prepared to a size of 2.5 × 5.0 cm size (30.0 ± 0.5 g) and placed in a polypropylene (PP) container (13.5 × 9.5 × 3.0 cm; 200 mL). To allow the indicator to react with the air in the headspace inside the container and result in a color change, a piece of GA/gelatin/Anth/PVA (2 × 2 cm) was attached to the interior of the PP film using tape, and the container was sealed (Figure S1). The beef samples were standardized to ensure uniformity in the meat marbling and the removal of excess fat from the oyster blade. For the squid samples, only the body sections after removing the fins and legs were used. The beef samples were stored at 4 and 25 °C, and the squid samples were stored at 4 °C. The pH and total volatile basic nitrogen (TVBN) were determined whenthe color change of the indicator was monitored to detect visible color changes for squid, and were measured on days 0, 2, 5, 7, 10, and 14 of 4 °C storage, and at 0, 5, 10, 24, and 50 h of 25 °C storage for beef. To measure the pH, the method described by Dirpan et al. [34] was followed. The beef samples (30.0 ± 0.5 g) were homogenized with DW (270 g) in a homogenizer (T25 digital ULTRA-TURRA; IKA, Staufen, Germany), and the pH of the homogenized solution was measured using a pH meter (Seven Compact S220; Mettler Toledo, Schwerzenbach, Switzerland).

For the TVBN analysis, the beef samples (30 g) were homogenized with 270 g DW [35], whereas the squid samples (30 g) were homogenized with 120 g of 4% trichloroacetic acid (TCA) solution for 4 min [36]. The homogenized solutions were filtered using Whatman No. 1 filter paper (pore size: 11 µm; Whatman International Limited, Kent, UK). The TVBN analysis was based on the Conway method described by Malle and Tao [37]. Initially, 1 mL of the beef or squid sample was placed in the outer chamber of the Conway dish, and 1 mL of 0.01 N boric acid was placed in the inner chamber. Following this, 100 µL of bromocresol green–methyl red solution and 1 mL of 50% K_2_CO_3_ were added to the inner and outer chambers, respectively. Then, the dish and lid were sealed using grease, and the reaction was allowed to proceed for 2 h at 37 °C. Subsequently, titration was performed using 0.01 N HCl until the color turned pink, and the amount of the consumed HCl (*a*) was recorded for the calculations (3). For the blank (*b*), DW and 4% TCA solution were used in lieu of the beef and squid samples, respectively, and titration was performed following the same procedure.

(3) $$TVBN\;\left(\mathrm{mg}/100\;g\right)=0.14\times \left(a-b\right)\times f\times d\times 100$$

where, *f* is the factor of 0.01 N HCl, and *d* is the dilution factor of the sample.

### 2.9. Statistical Analyses

All the experiments were conducted in duplicate. For the analysis of color stability against light, three samples of each film type—Anth/PVA, Gelatin/Anth/PVA, GA/Anth/PVA, GA/gelatin/Anth/PVA, AL/gelatin/Anth/PVA and AL/GA/gelatin/Anth/PVA—were prepared in each experiment (*n* = 6). Similarly, for the analysis of pH-dependent color change, determination of the LOD and LOQ against volatile amines, and assessment of the effects of time, temperature, and RH on pH indication, three GA/gelatin/Anth/PVA films were used in each experiment (*n* = 6). For the SEM, FTIR, and TGA analyses, one sample each of the PVA film, Anth/PVA, and GA/gelatin/Anth/PVA was prepared. Additionally, for the analyses of tensile properties and water solubility, seven and three samples of the PVA film, Anth/PVA, and GA/gelatin/Anth/PVA, respectively, were prepared in each experiment. Regarding the freshness monitoring of beef and squid, two PP containers, each containing the food and two attached indicators, were prepared on each day of analysis in each experiment. In all the experiments, the color of the indicator was measured five times. To compare the mean values from each experiment, one-way analysis of variance (ANOVA; Ver. 18, SPSS Inc., Chicago, IL, USA) with Tukey’s multiple-range test as post hoc test (α = 0.05) was used.

## 3. Results

### 3.1. Color Stability of pH Indicator Films Against Fluorescent Light

**Figure 1:**
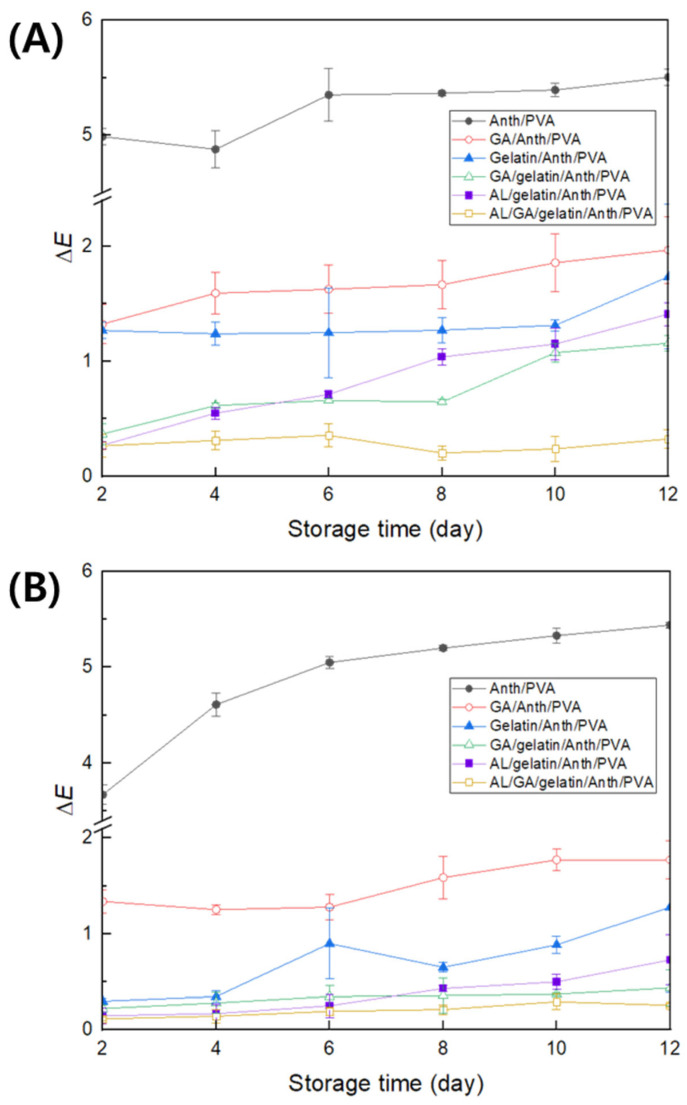
Changes in ∆*E* values of polyvinyl alcohol (PVA)-based pH-responsive color indicators using anthocyanin (Anth; Anth/PVA), Anth copigmented with gallic acid (GA; GA/Anth/PVA), Anth copigmented with gelatin (Gelatin/Anth/PVA), Anth copigmented with gelatin and GA (GA/gelatin/Anth/PVA), Anth copigmented with gelatin and aluminum ions (AL; AL/gelatin/Anth/PVA), and Anth copigmented with gelatin, GA, and AL (AL/GA/gelatin/Anth/PVA) determined during storage for 12 days at 4 °C (**A**) under fluorescent light and (**B**) dark conditions (*n* = 6).

Films with lower ∆*E* values are more color-stable, which is critical for color indication using a colorimetric pH indicator [38]. During the 12-day storage of Anth/PVA, Gelatin/Anth/PVA, GA/Anth/PVA, GA/gelatin/Anth/PVA, AL/gelatin/Anth/PVA, and AL/GA/gelatin/Anth/PVA at 4 °C under fluorescent-lamp and dark-room conditions, changes in ∆*E* were measured (Figure 1). The ∆*E* values on day 12 of storage under the fluorescent-lamp condition were 5.50 ± 0.07, 1.74 ± 0.63, 1.97 ± 0.29, 1.16 ± 0.07, 1.41 ± 0.10, and 0.32 ± 0.08 for Anth/PVA, Gelatin/Anth/PVA, GA/Anth/PVA, GA/gelatin/Anth/PVA, AL/gelatin/Anth/PVA, and AL/GA/gelatin/Anth/PVA, respectively. The corresponding values under the dark-room condition were 5.44 ± 0.03, 1.28 ± 0.89, 1.77 ± 0.2, 0.44 ± 0.19, 0.73 ± 0.26, and 0.26 ± 0.02, respectively. Under both conditions, the color stability was the highest for AL/GA/gelatin/Anth/PVA, followed by that for GA/gelatin/Anth/PVA, AL/gelatin/Anth/PVA, Gelatin/Anth/PVA, GA/Anth/PVA, and Anth/PVA.

The copigmentation process involving gelatin is facilitated by electrostatic interactions [16,39] and by the formation of hydrogen bonds between the carboxylate group (COO-) of gelatin and flavylium cations of Anths [17]. Copigmentation by GA can occur through π–π stacking interactions between the aromatic rings of GA and Anth [40]. Simultaneously, hydrogen bonding or charge-transfer interactions may arise between the hydroxyl group of GA, a type of hydroxybenzoic acid, and the carbonyl or hydroxyl groups of Anth [41]. Zhang et al. [42] reported that hydroxybenzoic acid, which contains more methoxyl or hydroxyl groups, interacts more effectively with malvidin-3-O-glucoside as the delocalization of π electrons in the copigment increases, leading to greater stabilization of Anth and enhancing π–π interactions between the aromatic rings and improving flavylium stabilization [43]. In this study, Gelatin/Anth/PVA exhibited greater stability compared with GA/Anth/PVA, which is likely to be because gelatin, unlike the low-molecular-weight compound GA, interacts with the high-molecular-weight polymer PVA, which serves as the film matrix, forming a robust structure that stabilizes Anth [44]. Metal-ion copigmentation occurs only with Anth containing catechol or pyrogallol groups in the B-ring, such as cyanidin, petunidin, delphinidin, and their derivatives. These compounds donate two electron pairs and simultaneously remove two protons to form an Anth–metal ion complex [19]. The red cabbage Anth used in this study consisted mainly of non-acylated or acylated derivatives of cyanidin-3-diglucoside-5-glucoside [45,46], and the addition of AL was expected to enhance the stability of Anth. However, a higher ∆*E* was observed in AL/gelatin/Anth/PVA than in GA/gelatin/Anth/PVA, which can be attributed to a substantial initial color change during storage as AL was added to non-acylated Anths, facilitating Anth oxidation [47]. Figure S2 illustrates the proposed mechanism of copigmentation for AL/GA/gelatin/Anth/PVA, which exhibited the highest Anth stability. The lowest ∆*E* value observed in AL/GA/gelatin/Anth/PVA can be attributed to the dual stabilization of Anth, where π–π stacking interactions occur between the flavylium ring (C-ring) of Anth and GA, whereas metal chelation takes place between the catechol ring (B-ring) of Anth and AL [48]. However, despite its superior stability, there are concerns regarding the potential transfer of AL to the food as AL/GA/gelatin/Anth/PVA is applied on the interior of packaging material, directly contacting the food. Thus, GA/gelatin/Anth/PVA, which exhibited the second highest stability against fluorescent light, was selected as the indicator suitable for food applications. It was used in subsequent experiments to assess stability, sensitivity, and R

### 3.2. pH-Dependent Color Change of the Anth Solution and GA/Gelatin/Anth/PVA

**Figure 2:**
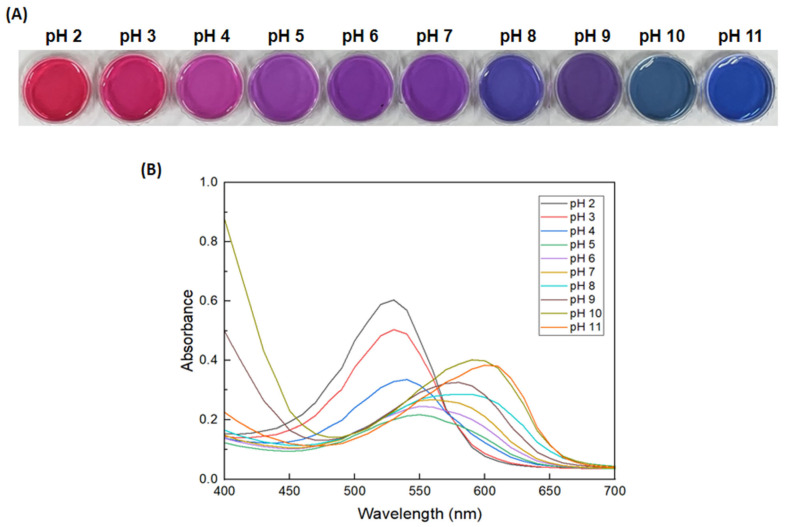
(**A**) Images and (**B**) UV–Vis spectra of the Anth solution in various pH buffer solutions.

Figure 2 presents the color changes and UV–Vis spectra of the Anth solution in various pH buffer solutions (pH 2–11). The Anth solution exhibited different colors depending on the pH: red at pH 2 and 3, purple at pH 4–7, and blue at pH 8–11. As the pH increased, the maximum absorption peak of the Anth solution shifted to longer wavelengths. At pH 2, the maximum absorption peak appeared at 530 nm, but as the pH increased to 5, the peak shifted to 550 nm, with a gradual decrease in absorbance. Between pH 5 and 11, the maximum absorption peak shifted from 550 nm to 600 nm as the pH increased, whereas the absorbance increased gradually. This indicates that a bathochromic shift (red shift), where the absorption peak shifts to longer wavelengths, occurred as the pH increased [49]. Table 1 presents the color and images of GA/gelatin/Anth/PVA in buffer solutions at various pH values. The indicator showed clear differences in color across varying pH levels; red at pH 2–4, purple at pH 5–7, and blue at pH 8–11, which is similar to that for the Anth solution shown in Figure 2. Anths exist as flavylium cations in acidic conditions, displaying a red hue. Upon deprotonation of the flavylium cations in neutral conditions, a neutral quinonoid base forms, resulting in a purple coloration. Further deprotonation to basic conditions results in a blue coloration that is attributable to the anionic quinonoid base [50]. The observed color changes of the Anth solution and GA/gelatin/Anth/PVA in the buffer solutions align with the pH-dependent color alterations of Anths. This distinct color modulation of the Anth solution and GA/gelatin/Anth/PVA based on the pH renders it suitable for evaluating the freshness of food items, as pH changes over time are indicative of spoilage.

### 3.3. Determination of LOD and LOQ of the GA/Gelatin/Anth/PVA Indicator Film

The calibration curves of the ∆*E* values for GA/gelatin/Anth/PVA exposed to various concentrations of TMA, DMA, and NH_3_, as well as the LOD and LOQ, are shown in Figure S3. The TMA, DMA, and NH_3_ solutions release TMA, DMA, and NH_3_ gases into the headspace inside the Petri dish through evaporation in aqueous form [51]. These gases then diffuse to the GA/gelatin/Anth/PVA film surface, where they react with H_2_O to form trimethylammonium ions ((CH_3_)_3_NH^+^), dimethylammonium ions ((CH_3_)_2_NH_2_^+^), and ammonium hydroxide (NH_4_^+^), along with hydroxide ions (OH^−^) [52]. The hydroxide ions deprotonate the Anth in the GA/gelatin/Anth/PVA film, causing a conversion from the flavylium ion form to the quinonoidal base and, eventually, to the anionic form, resulting in a visible color change in the GA/gelatin/Anth/PVA film [53]. The *R*^2^ values for the ∆*E* calibration curves of GA/gelatin/Anth/PVA after exposure to TMA, DMA, and NH_3_ were 0.99, 0.93, and 0.99, respectively. This indicated that the ∆*E* values of GA/gelatin/Anth/PVA showed high linearity according to the concentration of TMA, DMA, or NH_3_; hence, the LOD and LOQ determined using the slope and standard deviation estimated from the calibration curve were found to be valid. At concentrations ≥ 60 mg/L, the ∆*E* of GA/gelatin/Anth/PVA for DMA and NH_3_ were 3.09 ± 0.08 and 4.77 ± 0.43, respectively. For TMA at concentrations ≥ 500 mg/L, the ∆*E* of GA/gelatin/Anth/PVA was 3.83 ± 0.35, indicating a visible color difference in the film. The LOD values were determined to be 133.19, 13.26, and 21.83 mg/L, respectively, for TMA, DMA, and NH_3_, whereas the LOQ values were 403.61, 40.19, and 66.16 mg/L, respectively. These findings suggest that the relative sensitivity of the film was in the order of DMA > NH_3_ > TMA. Consequently, GA/gelatin/Anth/PVA exhibited color changes in the presence of TMA, DMA, and NH_3_, indicating its suitability for use in foods where DMA or NH_3_ is generated during spoilage rather than TMA.

### 3.4. Effects of Environmental Variables on GA/Gelatin/Anth/PVA

**Figure 3:**
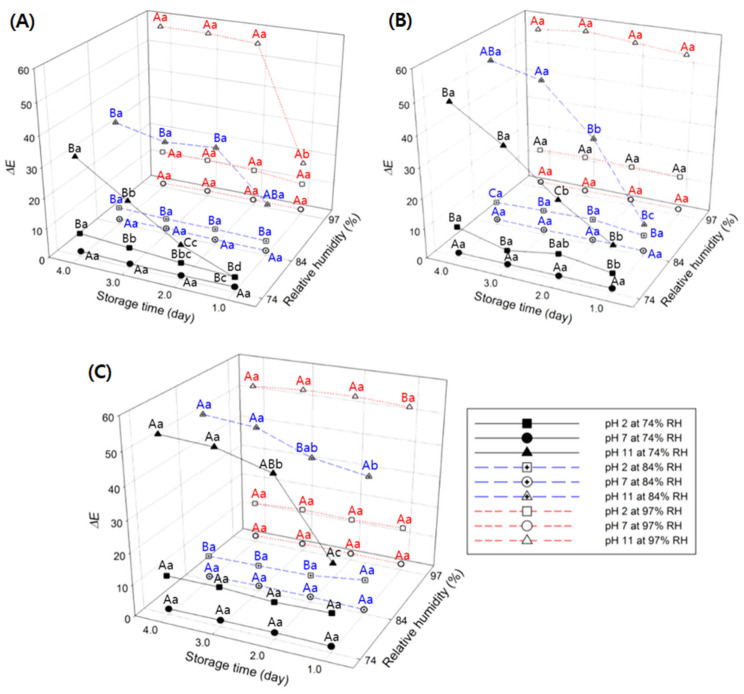
Changes in ∆*E* values of the polyvinyl alcohol (PVA)-based pH-responsive color indicator using anthocyanin (Anth) copigmented with gelatin and gallic acid (GA; GA/gelatin/Anth/PVA) at pH 2, 7, and 11 and 74, 84, and 97% RH with respect to storage time during storage at (**A**) 4 °C, (**B**) 10 °C, and (**C**) 25 °C. Means followed by different small letters (a–d) above the symbols indicate significant differences over storage time (*p* < 0.05), reflecting that the color changes of the indicators at the same pH level vary over time at the same RH. Means (*n* = 6) followed by different capital letters (A–C) above the symbols indicate significant differences over relative humidity (*p* < 0.05), illustrating the impact of varying RH levels on the color changes of the indicators at the same storage time point for each pH level.

**Figure 4:**
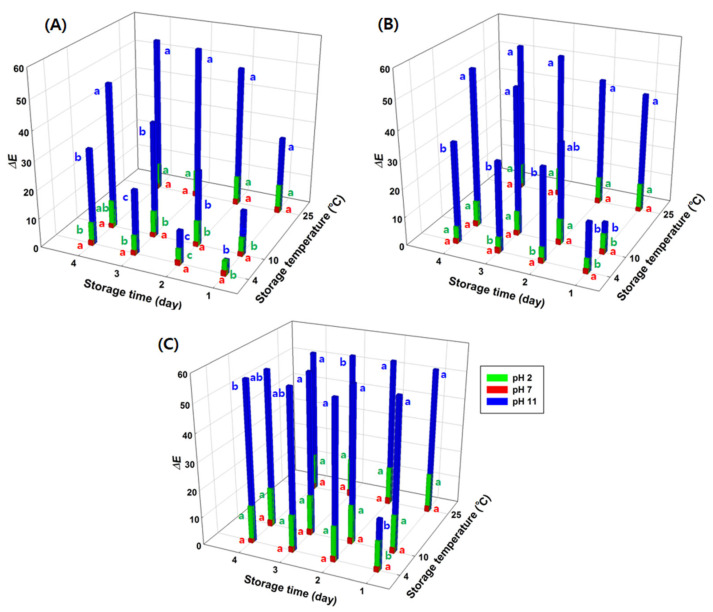
Changes in ∆*E* values of the polyvinyl alcohol (PVA)-based pH-responsive color indicator using anthocyanin (Anth) copigmented with gelatin and gallic acid (GA; GA/gelatin/Anth/PVA) at pH 2 and 7 at 4, 10, and 25 °C with respect to storage temperature during storage at (**A**) 74%, (**B**) 84%, and (**C**) 97% RH. Means (*n* = 6) followed by different small letters (a–c) above the symbols indicate significant differences over storage temperature (*p* < 0.05), reflecting the impact of varying temperatures on the color changes of the indicators at the same storage time point for each pH level.

The effects of RH and temperature on the pH indication by GA/gelatin/Anth/PVA during storage are shown in Figure 3 and Figure 4, respectively. The ∆*E* values of GA/gelatin/Anth/PVA at pH 7 did not significantly vary during storage at varying levels of RH at each temperature (*p* > 0.05). Under the temperature (4, 10, and 25 °C) and RH (74, 84, and 97%) conditions used in this study, the indicated stability of GA/gelatin/Anth/PVA at pH 7 was found to be high, as the color of the indicator at pH 7 was consistent during storage (4 days). At pH 2, the ∆*E* values did not significantly vary with storage time under all the storage conditions except for 4 °C/74% RH and 10 °C/74% RH (*p* > 0.05). Conversely, for pH 11, the ∆*E* values increased over time under all the storage conditions except for 4 °C/84% RH, 10 °C/97% RH, and 25 °C/97% RH (*p* < 0.05). The result suggests that at pH 2 and 7, GA/gelatin/Anth/PVA ensures relatively stable indication at all temperatures during storage, irrespective of time, compared with that at pH 11. The Anths in GA/gelatin/Anth/PVA at pH 11 are presumed to function as electron donors with an anionic chalcone structure [45], leading to rapid oxidation and, consequently, lower chemical stability than that at pH 2, with the flavylium cation structure, and pH 7, with the neutral quinonoid base structure. Thus, GA/gelatin/Anth/PVA is likely to be unsuitable for use as a freshness indicator for foods with alkaline pH ranges.

The ∆*E* values of GA/gelatin/Anth/PVA at pH 7 did not exhibit significant variation during storage at each time and temperature across the different RH levels (*p* > 0.05). Conversely, at pH 2, the ∆*E* values did not significantly differ according to the RH at conditions of 25 °C at storage days 1, 3, and 4 (*p* > 0.05); the ∆*E* values were larger at 97% RH than at 74% or 84% for all the other sets of storage time and temperature conditions (4 °C/storage days 1, 2, 3, 4; 10 °C/storage days 1, 2, 3, 4; and 25 °C/storage day 2) (*p* < 0.05). The structural instability of Anths at high RH levels under low pH conditions was consistent with the findings of Shin et al. [54], who found that the Anth content in strawberries stored at 10 °C (pH 3.0–4.0) decreased to a greater extent at 95% than at 65% RH.

The ∆*E* values of GA/gelatin/Anth/PVA at pH 7 did not significantly vary during storage at each time and RH across the different temperatures (*p* > 0.05). At pH 2, while the ∆*E* values did not significantly differ according to RH in the conditions of 84% RH/storage day 4 and 97% RH/storage days 1, 2, 3, and 4, respectively (*p* > 0.05), the ∆*E* values were the largest at 25 °C compared with those at other RH and storage time conditions (74% RH/storage days 1 2, 3, and 4 and 84% RH/storage days 1, 2, and 3) (*p* < 0.05). The impact of storage temperature on the rate of Anth degradation reportedly varies greatly according to the Anth type [55]. For the red cabbage Anths used in this study, the impact of storage temperature (4, 10, and 25 °C) on the structural stability of the Anths was influenced by the pH to which Anths were exposed, and at neutral pH, the impact was not perceptible.

The results showed that the indication stability of GA/gelatin/Anth/PVA at pH 7 remained consistently high across varying RH levels, whereas the stability of the indicator at pH 2 was notably robust only under conditions of elevated RH levels with changes in temperature. These findings collectively suggested that the most stable color could be obtained for acidic and neutral GA/gelatin/Anth/PVA indicators under high RH conditions. Consequently, the indicators developed in this study were conjectured to be suitable for foods at acidic or neutral pH ranges with high water activity. Thus, in this work, the selected foods for testing the applicability of these novel indicators were raw beef and raw squid, given their respective water activity levels of 0.98–0.99 [56] and 0.83–0.84 [57], as well as their indicative pH ranges for freshness to spoilage of 5.58–6.00 [58] and 6.66–7.42 [59], respectively.

### 3.5. Film Characterization

**Figure 5:**
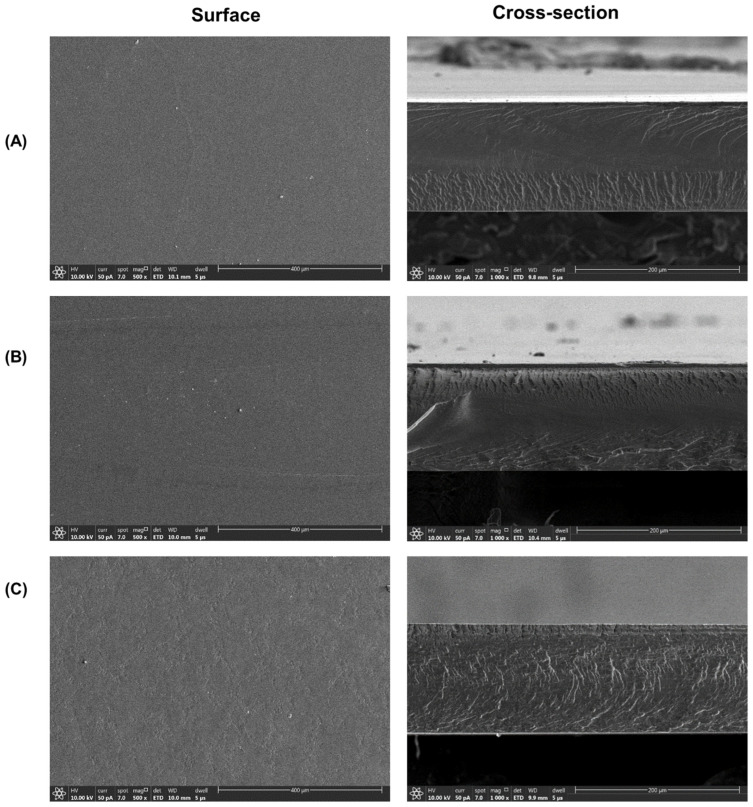
Scanning electron microscopy images of the surfaces and cross-sections of the (**A**) polyvinyl alcohol (PVA) film, (**B**) PVA-based pH-responsive color indicator using anthocyanin (Anth; Anth/PVA), and (**C**) PVA-based pH-responsive color indicator using Anth copigmented with gelatin and gallic acid(GA; GA/gelatin/Anth/PVA).

Figure 5 shows SEM images, revealing the surface and cross-sections of the PVA film, Anth/PVA, and GA/gelatin/Anth/PVA. The PVA film and Anth/PVA exhibited smooth surfaces and cross-sections, whereas GA/gelatin/Anth/PVA presented a rougher texture in both the surface and cross-sectional views than did the PVA film and Anth/PVA. In particular, microcraters formed on the surface of GA/gelatin/Anth/PVA due to phase segregation resulting from the interactions between PVA and gelatin, which are influenced by their different ratios [60]. During the film-drying process, rapid water evaporation leads to the formation of small droplets on the film surface due to surface tension. The phase segregation between PVA and gelatin causes gelatin to migrate toward the edges of these droplets, resulting in microcraters after the water evaporates [61]. This disparity was attributed to the formation of a complex network within the film through hydrogen bonding or electrostatic interactions between PVA and gelatin, which likely caused the irregularities in the structure, thus leading to the roughness observed in the cross-sections [62].

**Figure 6:**
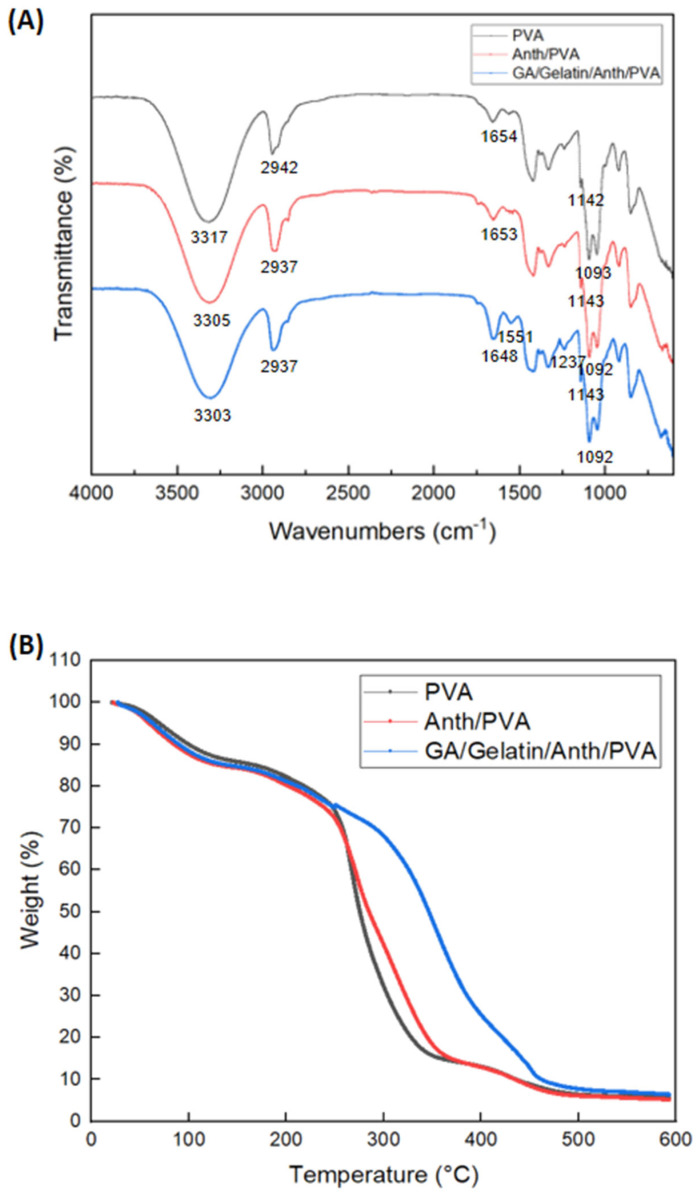
(**A**) Fourier-transform infrared spectroscopy spectra and (**B**) thermal degradation curves of the polyvinyl alcohol (PVA) film, PVA-based pH-responsive color indicator using anthocyanin (Anth; Anth/PVA), and polyvinyl alcohol-based pH-responsive color indicator using Anth copigmented with gelatin and gallic acid (GA; GA/gelatin/Anth/PVA).

Figure 6A shows the surface chemical structures of the PVA film, Anth/PVA, and GA/gelatin/Anth/PVA obtained using FTIR analysis. All three samples shared characteristic peaks of PVA at various wavenumbers: around 3300 cm^−1^ for O-H stretching, around 2940 cm^−1^ for C-H stretching, around 1650 cm^−1^ for C=O stretching, around 1140 cm^−1^ for C-C and C-O stretching, and around 1090 cm^−1^ for C-O stretching and O-H bending [63]. In the case of Anth/PVA, the intermolecular hydrogen bonding between Anth and PVA led to the peak shift from 3317 cm^−1^ to a lower frequency at 3305 cm^−1^, and the peak intensity of the O-H stretching was reduced. Similarly, the addition of gelatin in GA/gelatin/Anth/PVA led to hydrogen bonding formation between Anth, PVA, and gelatin, resulting in a peak shift to 3303 cm^−1^ and reduced peak intensity [64].

Compared with the PVA film and Anth/PVA, GA/gelatin/Anth/PVA showed more distinct peak values at 1648 and 1237 cm^−1^, presumably due to the formation of an amide I band (C=O stretching vibration) and amide III band (CN stretching vibration), respectively, as a result of the gelatin addition [65]. The peak at 1237 cm^−1^, corresponding to the C-O stretching vibration, also indicates the presence of GA [66]. Additionally, the increased peak value at 1551 cm^−1^ for GA/gelatin/Anth/PVA, compared with that for PVA and Anth/PVA, could be attributed to the formation of an amide II band (NH stretching) as a result of the gelatin addition and the vibration from the stretching of the C=C aromatic ring due to the addition of GA. The ester bonding between Anth and GA in GA/gelatin/Anth/PVA was confirmed based on the peak indicating the C-O-C stretching vibration at 1237 cm^−1^ [67]. The stretching of the C=C aromatic ring suggests that the interaction between Anth and GA is influenced by noncovalent forces, such as π–π stacking interactions [68].

Figure 6B presents the thermal stability of the PVA film, Anth/PVA, and GA/gelatin/Anth/PVA. All three films showed weight loss caused by the evaporation of water from the film interior at temperatures ranging from approximately 50 to 120 °C. The molecular denaturation of PVA occurred at approximately 240–350 °C for the PVA film and Anth/PVA and at approximately 240–450 °C for GA/gelatin/Anth/PVA, which likely contributes to the second weight loss observed [69]. Notably, GA/gelatin/Anth/PVA exhibited delayed weight loss compared with the PVA film and Anth/PVA, which was attributed to the increased thermal stability of GA/gelatin/Anth/PVA through the hydrogen bonding between PVA and gelatin [70]. This enhanced thermal stability was corroborated by the lower peak value observed at 3303 cm^−1^, indicating the presence of hydrogen bonding between PVA and gelatin in the FTIR analysis of GA/gelatin/Anth/PVA (Figure 6A).

Table 2 demonstrates the measured tensile properties of the PVA film, Anth/PVA, and GA/gelatin/Anth/PVA. The tensile strength, elastic modulus, and elongation at break did not significantly vary across the three films (*p* > 0.05). The addition of gelatin could lead to a decreasing trend in elongation at break with increased tensile strength due to hydrogen bonding or electrostatic interactions between PVA and gelatin [71]; however, the amount of gelatin used in this study was believed to be insufficient to significantly affect the tensile properties. Therefore, the incorporation of Anth with PVA and copigmentation did not appear to influence the strength, stretchability, or brittleness of PVA.

Table 2 also provides data on the water solubility of the PVA film, Anth/PVA, and GA/gelatin/Anth/PVA. Compared with the PVA film and Anth/PVA, GA/gelatin/Anth/PVA had higher water solubility (*p* < 0.05), presumably because of the higher hydrophilic property of gelatin. This increase in water solubility with the addition of gelatin is in agreement with the observations of Jridi et al. [72], who demonstrated an increase in the solubility of a composite film made up of chitosan and gelatin with an increase in the proportion of gelatin.

Regarding the earlier suggestion of the applicability of GA/gelatin/Anth/PVA in foods with high water activity, it should be noted that care should be exercised, as there is a possibility that the indicator may dissolve in foods with high water activity during long-term storage.

### 3.6. Freshness Monitoring of Beef and Squid Using GA/Gelatin/Anth/PVA

**Figure 7:**
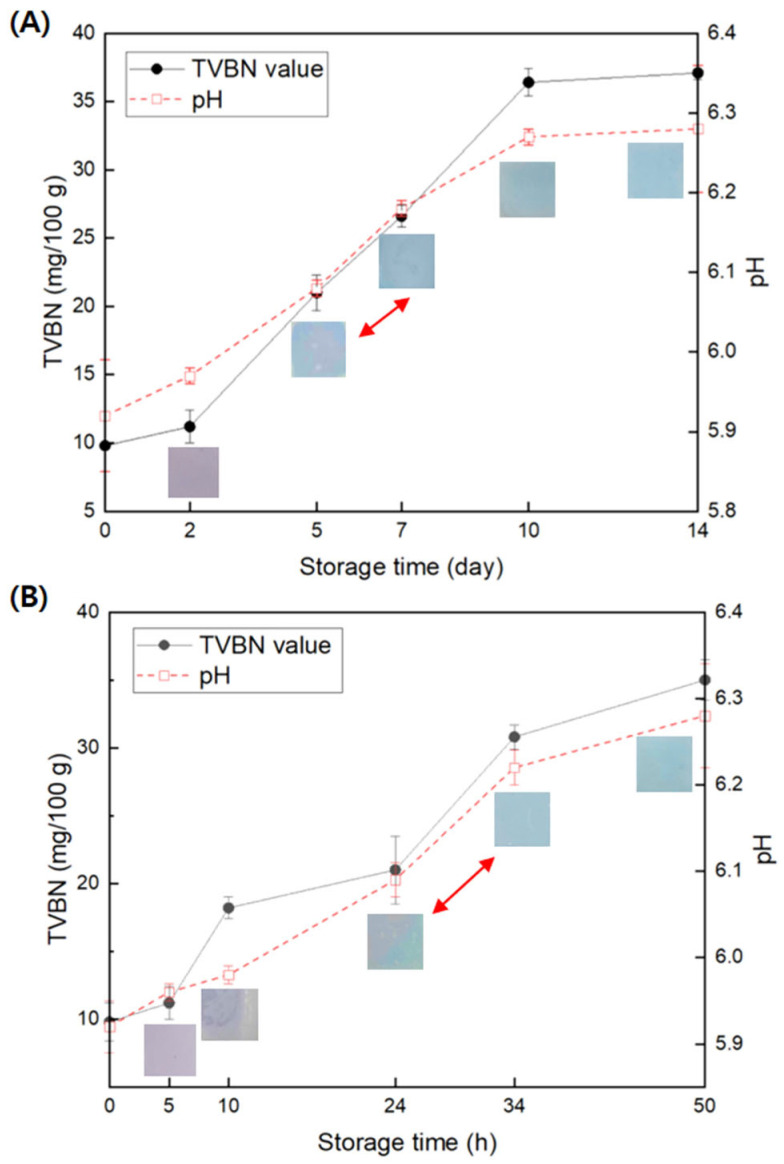
Changes in the pH and TVBN values (*n* = 4) of raw beef and the color of the polyvinyl alcohol (PVA)-based pH-responsive color indicator using anthocyanin (Anth) copigmented with gelatin and gallic acid (GA; GA/gelatin/Anth/PVA) during storage at (**A**) 4 and (**B**) 25 °C.

For the beef stored at 4 and 25 °C, the color of the GA/gelatin/Anth/PVA and the TVBN and pH values of the raw beef according to storage time are shown in Figure 7. Over the 14-day storage period at 4 °C, the pH and TVBN of the film increased from 5.97 ± 0.07 to 6.28 ± 0.08 mg/100 g and from 9.8 ± 0.2 to 37.1 ± 0.5 mg/100 g, respectively. Likewise, during the storage of beef at 25 °C over 50 h, the pH and TVBN of the film steadily increased from 5.96 ± 0.03 to 6.28 ± 0.06 mg/100 g and from 9.8 ± 1.4 to 35.0 ± 1.5 mg/100 g, respectively. The color of the indicator transitioned from purple to a combination of purple and blue by day 5 of storage at 4 °C, ultimately turning completely blue by day 7 (Figure 7A). In contrast, during storage at 25 °C, the color of the indicator remained purple for the first 10 h, shifted to a combination of purple and blue at 24 h, and turned completely blue at 34 h (Figure 7B). The TVBN levels at the onset of the color change (4 °C/day 5 and 25 °C/24 h) were 21.0 ± 1.3 and 21.0 ± 2.5 mg/100 g, respectively, aligning with the threshold for edible meat spoilage at 20.0 mg/100 g [73]. These results demonstrate the effectiveness of GA/gelatin/Anth/PVA in detecting the onset of beef spoilage, affirming its potential as a freshness indicator for the refrigerated fresh meat sold in small packages at the supermarket.

The applicability of the indicator for assessing squid freshness was also examined. The TVBN on day 7, when the indicator exhibited a blue color during squid storage, was 37.8 ± 2.4 mg/100 g, which is consistent with the threshold for seafood spoilage at 35 mg/100 g [74] (Figure S1B). Therefore, the novel indicator demonstrates applicability in indicating squid freshness.

At the point where the indicator color transitioned completely from light blue to purple during storage of the beef (4 °C/day 7 and 25 °C/34 h), the pH of the beef was 6.18 ± 0.01 and 6.22 ± 0.02, respectively. However, the color of the indicator remained purple when reacting to a buffer solution at a similar pH (pH 6) (Table 1). This discrepancy suggests that the change in color of the indicator does not directly mirror the pH of the beef. Instead, it is the amines generated during beef spoilage over time that react with water molecules on the indicator surface, leading to the generation of OH^−^ ions and the subsequent color change [75].

## 4. Conclusions

In this study, we used Anth as the pH-indicating material, PVA as the indicator base, and gelatin, GA, and AL as copigments in developing novel freshness indicators. Among these, the GA/gelatin/Anth/PVA indicator, incorporating two copigments, exhibited high stability against light. GA/gelatin/Anth/PVA showed the highest sensitivity for DMA. As GA/gelatin/Anth/PVA led to a stable color change based on the pH in acidic and neutral foods with high water activity, food with high water activity and pH values in acidic–neutral ranges upon spoilage were determined to be suitable for the application of these novel indicators. The addition of gelatin and GA to Anth/PVA led to the formation of hydrogen bonds between Anth and gelatin, as well as between Anth and GA. This led to increased roughness of the surface and cross-sections, enhanced thermal stability, and elevated water solubility, while the tensile properties remained largely unaffected. The GA/gelatin/Anth/PVA indicator effectively signaled the spoilage of beef and squid, as evidenced by the agreement in TVBN levels with the color change of the indicator and established TVBN criteria for food spoilage. These results support the potential utility of GA/gelatin/Anth/PVA as a reliable freshness indicator for raw beef and squid. Nonetheless, identifying the color displayed in the indicator can be difficult, because it is affected by light conditions and consumer fatigue, and the color can be misread by consumers. A mobile application that identifies the color of the indicators using deep learning technology can be applied to judge the color objectively without misreading.

## Figures and Tables

**Table 1:** Chromaticity values and images of the polyvinyl alcohol (PVA)-based pH-responsive color indicator using anthocyanin (Anth) copigmented with gelatin and gallic acid (GA; GA/gelatin/Anth/PVA) in pH buffer solutions at different pH levels.

pH of Buffer Solutions	2	3	4	5	6	7	8	9	10	11
GA/Gelatin/Anth/PVA Images	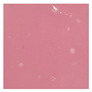	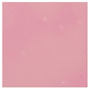	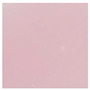	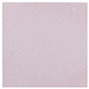	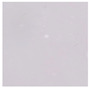	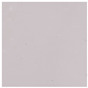	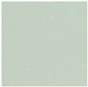	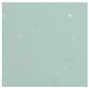	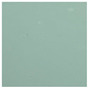	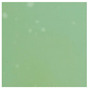
L*	82.21 ± 0.21 d	84.16 ± 0.34 c	86.85 ± 0.18 b	88.06 ± 0.12 a	87.86 ± 0.33 a	88.02 ± 0.20 a	87.71 ± 0.22 a	87.70 ± 0.11 a	87.70 ± 0.11 a	87.70 ± 0.11 a
a*	19.50 ± 0.35 a	14.76 ± 0.55 b	7.50 ± 0.13 c	3.58 ± 0.08 d	3.15 ± 0.26 e	1.76 ± 0.29 f	−0.09 ± 0.13 g	−1.68 ± 0.31 h	−2.96 ± 0.17 i	−7.79 ± 0.27 j
b*	1.56 ± 0.05 ef	1.23 ± 0.10 ef	1.82 ± 0.03 de	2.37 ± 0.04 b	2.24 ± 0.07 bc	2.18 ± 0.03 bcd	−0.99 ± 0.13 cde	2.20 ± 0.04 bcd	1.94 ± 0.06 cde	7.06 ± 0.55 a

Data are expressed as the mean ± standard deviation (*n* = 6). Values followed by different letters in the same row are significantly different based on Tukey’s test (*p* < 0.05).

**Table 2:** Tensile properties and water solubility of the polyvinyl alcohol (PVA) film, PVA-based pH-responsive color indicator using anthocyanin (Anth; Anth/PVA), and PVA-based pH-responsive color indicator using Anth copigmented with gelatin and gallic acid (GA; GA/gelatin/Anth/PVA).

Film	PVA Film	Anth/PVA	GA/Gelatin/Anth/PVA
Tensile strength (MPa)	22.98 ± 2.07 a	21.21 ± 3.58 a	20.52 ± 3.30 a
Elongation at break (%)	466.54 ± 0.86 a	466.31 ± 0.43 a	466.38 ± 0.16 a
Elastic modulus	55.83 ± 4.20 a	53.08 ± 2.77 a	57.24 ± 8.44 a
Water solubility (%)	8.71 ± 2.12 b	7.78 ± 2.68 b	13.88 ± 0.90 a

Data are expressed as the mean ± standard deviation. Values followed by different letters in the same row are significantly different based on Tukey’s test (*p* < 0.05).

## Data Availability

The original contributions presented in the study are included in the article and Supplementary Materials, further inquiries can be directed to the corresponding author.

## Supplementary Materials

The following supporting information can be downloaded at: https://https://www.mdpi.com/article/10.3390/foods13213464/s1, Figure S1: Application of a polyvinyl alcohol (PVA)-based pH-responsive color indicator using anthocyanin (Anth) copigmented with gelatin and gallic acid (GA; GA/gelatin/Anth/PVA) inside a polypropylene container (lid) containing (**A**) raw beef and (**B**) squid; Figure S2: Schematic drawing illustrating the copigmentation of aluminum ion (AL), gallic acid (GA), and gelatin on anthocyanin (Anth) in a polyvinyl alcohol (PVA)-based pH-responsive color indicator using Anth copigmented with gelatin, GA, and AL; Figure S3: Linear correlations between Δ*E* values of a polyvinyl alcohol (PVA)-based pH-responsive color indicator using anthocyanin (Anth) copigmented with gelatin and gallic acid (GA; GA/gelatin/Anth/PVA) and the tested concentration of (**A**) trimethylamine (TMA), (**B**) dimethylamine (DMA), and (**C**) NH_3_ exposed to the indicator.
