# Supplementary material for: Monitoring Meat Freshness with Intelligent Colorimetric Labels Containing Red Cabbage Anthocyanins Copigmented with Gelatin and Gallic Acid
Source: Foods. 2024 Oct 29;13(21):3464. doi: 10.3390/foods13213464 (PMC11545453; doi:10.3390/foods13213464)
Supplement: Supplementary file 1 [file foods-13-03464-s001.zip › foods-3250659-supplementary.pdf]

Monitoring Meat Freshness with Intelligent Colorimetric Labels Containing Red Cabbage Anthocyanins Copigmented with Gelatin and Gallic Acid

Minyoung Kwak [1] and Sea C. Min [1]*

[1] Department of Food Science and Technology, Seoul Women's University, 621, Hwarangro, Nowon-gu, Seoul, 01797, Republic of Korea; 3in0e@naver.com (M.Y.K.)
* Correspondence: smin@swu.ac.kr; Tel.: +82-2-970-5635 (S.C.M.)

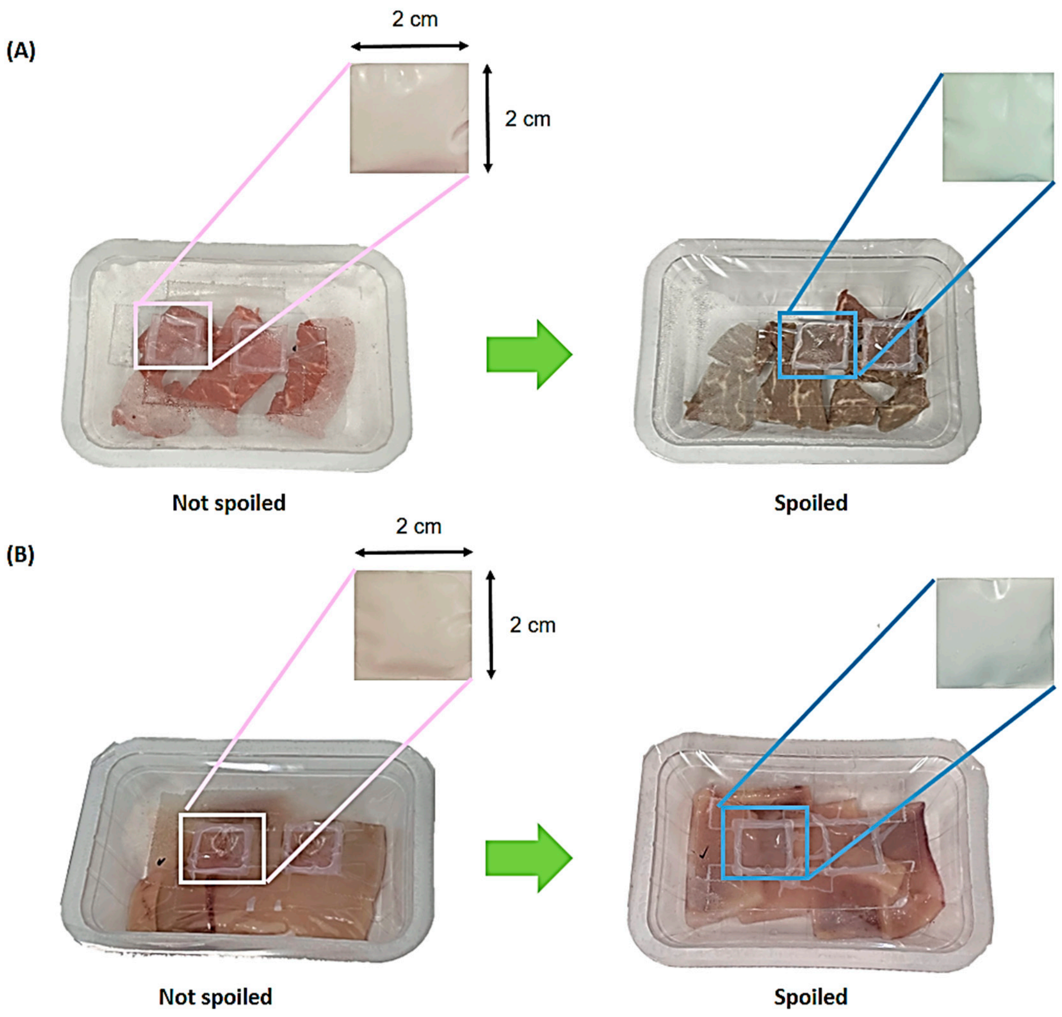

Figure S1. Application of polyvinyl alcohol (PVA)-based pH-responsive color indicator using anthocyanin (Anth) copigmented with gelatin and gallic acid (GA; GA/gelatin/Anth/PVA) inside a polypropylene container (lid) containing (A) raw beef and (B) squid.

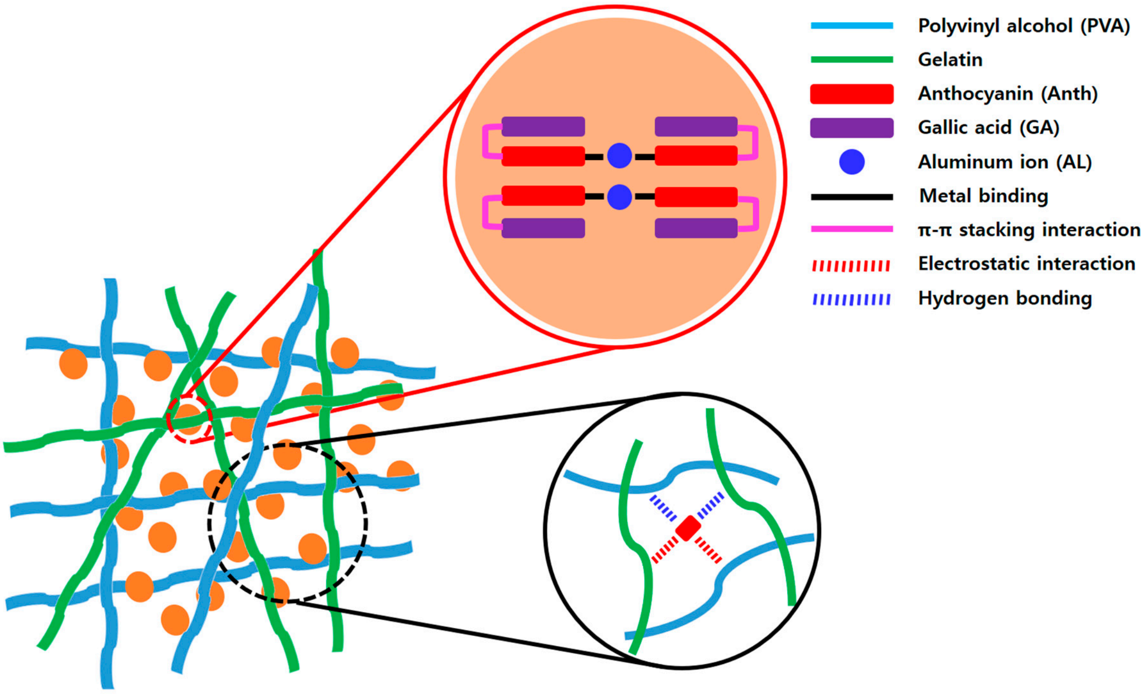

Figure S2. Schematic drawing illustrating the copigmentation of aluminum ion, gallic acid (GA), and gelatin on anthocyanin (Anth) in polyvinyl alcohol (PVA)-based pH-responsive color indicator using Anth copigmented with gelatin, GA, aluminum ion (AL; AL/GA/gelatin/Anth/PVA).

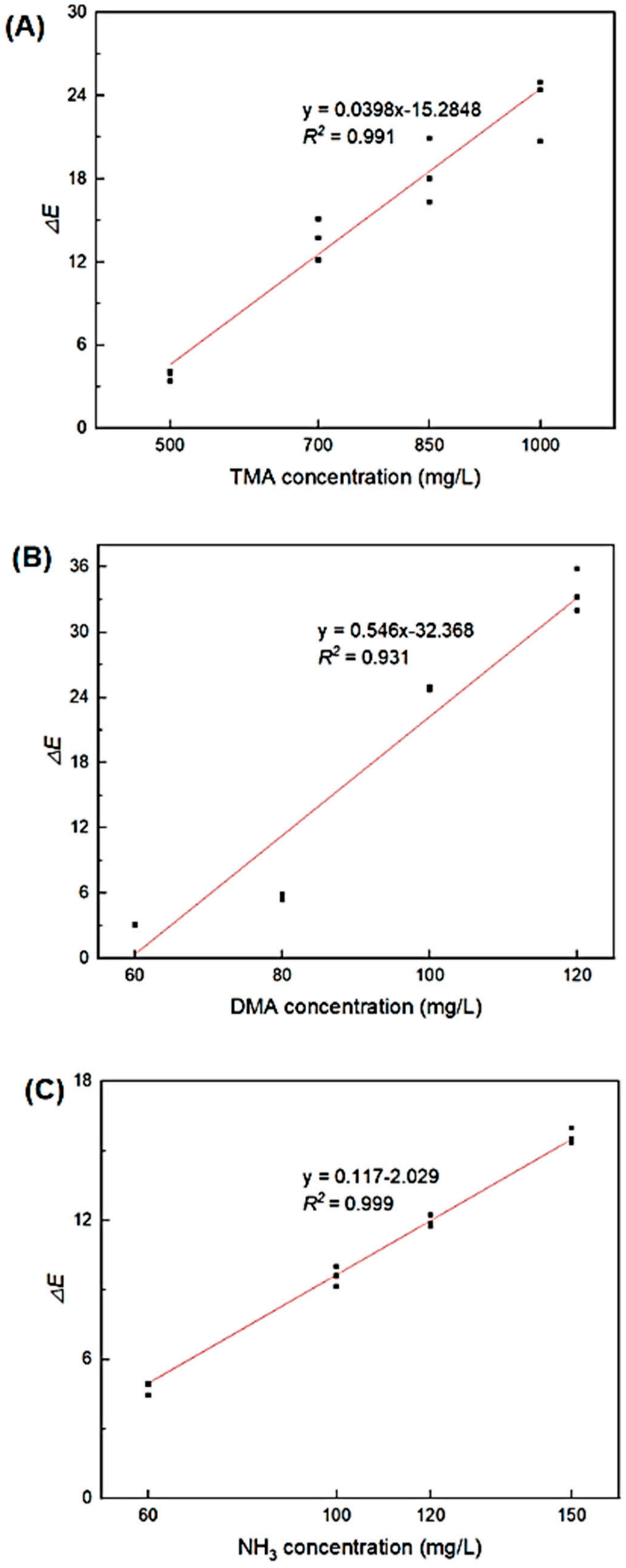

Figure S3. Linear correlations between *ΔE* values of polyvinyl alcohol (PVA)-based pH-responsive color indicator using anthocyanin (Anth) copigmented with gelatin and gallic acid (GA; GA/gelatin/Anth/PVA) and the concentration of (A) trimethylamine (TMA), (B) dimethylamine (DMA), and (C) NH_3 exposed to the indicator.
